# Genome Sequence of Bacterial Interference Strain *Staphylococcus aureus* 502A

**DOI:** 10.1128/genomeA.00284-14

**Published:** 2014-04-10

**Authors:** Dane Parker, Apurva Narechania, Robert Sebra, Gintaras Deikus, Samuel LaRussa, Chanelle Ryan, Hannah Smith, Alice Prince, Barun Mathema, Adam J. Ratner, Barry Kreiswirth, Paul J. Planet

**Affiliations:** a Columbia University, New York, New York, USA; b Sackler Institute for Comparative Genomics, American Museum of Natural History, New York, New York, USA; c Genome Center, Mount Sinai Hospital, New York, New York, USA; d Public Health Research Institute, Rutgers University, Newark, New Jersey, USA

## Abstract

*Staphylococcus aureus* 502A was a strain used in bacterial interference programs during the 1960s and early 1970s. Infants were deliberately colonized with 502A with the goal of preventing colonization with more invasive strains. We present the completed genome sequence of this organism.

## GENOME ANNOUNCEMENT

*Staphylococcus aureus* 502A was initially isolated in 1963 from a nurse in a newborn nursery who was caring for a cluster of 40 neonates who also became colonized with this strain (1). Colonization with 502A appeared to prevent infection with the exfoliative toxin-producing strain 80/81 that was epidemic in hospitals during the early 1960s (1–4). Thousands of neonates were inoculated with 502A in their nares and on their umbilical stumps, and the strain often persisted for months, only rarely causing disease itself (3, 5, 6). Strain 502A was also effective in protecting adults from virulent isolates of *S. aureus* (2, 7). However, the practice was abandoned after several infections and a death were attributed to the 502A strain (5). We recently showed that the innate immune response to 502A is markedly different from the response to the current epidemic USA300 strain, but our initial genomic analysis did not find any striking differences in the repertoire of virulence determinants (8).

*S. aureus* DNA was isolated after lysostaphin treatment (6 µg/ml, 1 h, 37°C) using standard procedures. We used 3.5 µg of DNA to construct SMRTbell libraries for the PacBio (Pacific Bioscience) sequencing platform using polymerase enzyme-DNA bound complexes with an average insert size of ~20 kb, as assessed by the Agilent 12000 bioanalyzer gel chip following size selection using the Sage BluePippin 0.75% agarose cassette from 7,000 to 50,000 bp. The binding chemistry was done using the PacBio P5-C3 DNA/polymerase binding kit. A DNA/polymerase complex of the sample was prepared using 0.5 nM of the SMRTbell library and 10× excess DNA polymerase. All 20-kb samples were magbead loaded prior to immobilization at 75 pM for 30 min on SMRTcells, ensuring Poisson-like loading distribution. Sequencing was done on a PacBio-RSII sequencer using 180-min continuous collection times and C3 sequencing chemistry, allowing collection of subreads of ~36,000 bp. We obtained 236× coverage, with 145,600 mapped reads with a mean read length of 6,401 bp (*N*_50_, 8,802 bp). Assembly of reads was done with the HGAP2 v 2.1 *de novo* assembly pipeline, generating five contigs.

Manual assembly resolved to 2 contigs, a circular chromosome (2,776,796 bp) and a plasmid (22,900 bp). The plasmid, designated SAP060A, had been sequenced previously (GQ900416.1) and has genes for putative enterotoxins and cadmium resistance. Comparison with a 502A Ion Torrent-generated genomic sequence (8) revealed only one high-quality (q > 20) base-pair difference, suggesting high accuracy. However, there were 888 insertions/deletions identified between sequences. This discrepancy may be due to known homopolymer-related problems in Ion Torrent- and PacBio-generated sequences.

Colonization is a prerequisite for invasive disease, making the factors of this initial step in infection excellent targets for prevention. The complete genome sequence of 502A may help develop strategies that favor colonization with relatively benign strains over more virulent organisms or selectively target virulent organisms for decolonization.

### Nucleotide sequence accession numbers.

This genome sequence has been deposited in GenBank under the accession numbers CP007454 and CP007455.
